# Editorial: Metabolic Mediators and Synapses: Linking Body Periphery to Neural Plasticity

**DOI:** 10.3389/fncel.2019.00378

**Published:** 2019-08-27

**Authors:** Margherita Maffei, Marco Mainardi

**Affiliations:** ^1^Institute of Clinical Physiology, National Research Council, Pisa, Italy; ^2^Endocrinology Unit, Obesity Center, University Hospital of Pisa, Pisa, Italy; ^3^Laboratory of Biology “Bio@SNS”, Scuola Normale Superiore, Pisa, Italy; ^4^Institute of Neuroscience, National Research Council, Pisa, Italy

**Keywords:** hypothalamus, hippocampus, learning and memory (neurosciences), hormone, gut, Alzheimer's disease, diet

Neural circuits are endowed with the unique capability to tailor their activity and connectivity in response to incoming stimuli, in order to orchestrate appropriate behavioral responses. The plethora of molecular and functional changes serving these adaptive processes is collectively referred to as “synaptic plasticity.” In addition to sensory stimuli, synaptic plasticity can be also triggered and modulated by internal messengers, epitomized by neurotrophins (Reichardt, 2006; Begni et al., 2017). Hormonal regulators of metabolism show similar properties. For instance, administration of leptin to *ob/ob* mice (a natural loss-of-function mutant for the *obese* gene, encoding leptin) induces rewiring of synaptic inputs onto hypothalamic neurons (Pinto et al., 2004). Strikingly, this action extends outside of nuclei involved in metabolic homeostasis; indeed, leptin treatment affects synaptic transmission and long-term potentiation in the hippocampus, the brain's hub for learning and memory (Mainardi et al., 2013). Analogous capabilities have also been observed for insulin, ghrelin and glucagon-like peptide-1 (GLP-1; Mainardi et al., 2015). On the other hand, the synaptic plasticity master controller Brain-Derived Neurotrophic Factor (BDNF) can be also considered a metabolic regulator, as it represses food intake via its action on the ventromedial hypothalamus (VMH; Xu et al., 2003). Interestingly, compounds that stimulate synaptic plasticity in cortical areas by increasing BDNF levels, such as fluoxetine (Maya Vetencourt et al., 2008), have similar effects on the hypothalamus (Barone et al., 2018), which can lead to enhanced leptin sensitivity via signaling mediated by TrkB, the main receptor for BDNF (Scabia et al., 2018).

Starting from these interesting functional parallels between metabolic hormones and neurotrophins, this Research Topic has the goal of providing an update on the rapidly developing field of the interaction between metabolism and brain plasticity.

Understanding how hormones interact with hypothalamic circuits is the primary target of research on brain-metabolism interactions. In this regard, dos-Santos et al. used multi-electrode array and patch-clamp recordings plus single-cell rtPCR to elucidate how ghrelin can regulate the activity of the paraventricular hypothalamic nucleus, leading to decreased secretion of TRH and CRH. Their results fit into the general picture delineated by Serrenho et al., who discuss the effects of ghrelin on synaptic plasticity of the integrated feeding behavior regulatory system, composed of brain regions controlling homeostasis, motivation and memory.

Metabolic hormones bind their receptors to trigger signal transduction processes that are, in principle, sufficient to support synthesis or modification of proteins involved in synaptic transmission. However, the balance of metabolically active molecules can *per se* affect neuronal activity, which can be applied to ameliorate pathological conditions, such as epilepsy (Testa et al., 2019). Within this context, Calì et al. summarize our current knowledge on the central role of L-lactate as the energetic exchange currency between astrocytes and neurons. Strikingly, the Authors show that L-lactate also acts as a signal that regulates synaptic plasticity (Calì et al.). We envisage that future research should take into account this fact by dissecting between actions of hormones centered on signaling and on regulation of energy substrate synthesis and availability.

An apparently secondary target of metabolic hormones is the hippocampus, the brain's hub for formation and recall of episodic memories. As discussed by McGregor and Harvey, leptin exerts neurotrophin-like actions on this structure, by enhancing synaptic plasticity and, in turn, learning and memory. The implications of this idea extend to brain pathology, namely age-related cognitive decline and Alzheimer's disease, whose severity can be exacerbated by leptin insufficiency or resistance (McGregor and Harvey). This hypothesis is in accordance with data showing that a hyperlipidic diet leads to impaired plasticity (Spinelli et al., 2017) and leptin resistance in the hippocampus (Mainardi et al., 2017).

Insulin also takes part in the enlarging network of interactions that maintains neural circuit homeostasis (Mainardi et al., 2015). Indeed, Gonçalves et al. provide an overview on the relationship between insulin and Tau protein, and on their implications for tauopathy and Alzheimer's disease. Accordingly, a dietary regimen centered on a high intake of fats is now recognized to negatively affect hippocampal function, as highlighted in the review by Del Olmo and Ruiz-Gayo.

Interestingly, the consequences of deficiency or resistance to metabolic hormones on learning and memory are accompanied by direct effects on basic homeostatic functions, such as feeding and circadian regulation, which, as Hiller and Ishii discuss, indicate that the hypothalamus is an early target of neurodegeneration. This concept could suggest fresh approaches to the early diagnosis and therapy of Alzheimer's disease and dementia (Hiller and Ishii) and, to further strengthen its potential, recent evidence indicates that the hypothalamus responds to stimuli that induce synaptic plasticity (Mainardi et al., 2010; Barone et al., 2018) also in the adult age.

An apparently straightforward development of all these findings is the design of therapies aimed at counteracting neurodegeneration by restoring metabolic hormone-associated signaling in the brain. Indeed, Sanguinetti et al. first demonstrate that a hyperlipidic diet worsens the severity of the phenotype in the 3xTg mouse model of Alzheimer's disease, and then that intranasal insulin treatment attenuates the signs of neurodegeneration, in addition to ameliorating metabolic dysregulation. However, metabolic hormones activate multiple signaling pathways with possible opposite effects on synaptopathy. Cisternas et al. suggest the feasibility of a more precise approach, by using a Wnt inhibitor, andrographolipide, whose administration to the J20 mouse AD model ameliorated both metabolic and cognitive aspects of the disease.

This collection of contributions supports the idea that metabolic hormones are key players in the delicate regulation of synaptic plasticity. Moreover, experimental work indicates that altered metabolic homeostasis is a strong determinant of the severity of age-related cognitive decline and neurodegeneration. In addition to prompting further research on the physiological aspects of the matter, we hope that this Research Topic will succeed in stressing the importance of harnessing the potential of metabolic hormones to counteract neurodegeneration and dementia.

## Author Contributions

MMai and MMaf discussed the manuscripts submitted to the Research Topic, the current literature, and wrote the manuscript.

### Conflict of Interest Statement

The authors declare that the research was conducted in the absence of any commercial or financial relationships that could be construed as a potential conflict of interest.
